# CAR-NK cells for cancer immunotherapy: from bench to bedside

**DOI:** 10.1186/s40364-022-00364-6

**Published:** 2022-03-18

**Authors:** Leisheng Zhang, Yuan Meng, Xiaoming Feng, Zhongchao Han

**Affiliations:** 1 Key Laboratory of Molecular Diagnostics and Precision Medicine for Surgical Oncology in Gansu Province & NHC Key Laboratory of Diagnosis and Therapy of Gastrointestinal Tumor, Gansu Provincial Hospital, Lanzhou, 730000 China; 2 Center for Cellular Therapies, The First Affiliated Hospital of Shandong First Medical University, Ji-nan, 250014 China; 3 Key Laboratory of Radiation Technology and Biophysics, Hefei Institute of Physical Science, Chinese Academy of Sciences, 350 Shushanhu Road, Shushan District, Hefei, 230031 Anhui Province China; 4 Institute of Stem Cells, Health-Biotech (Tianjin) Stem Cell Research Institute Co., Ltd, Tianjin, 301700 China; 5 Jiangxi Research Center of Stem Cell Engineering, Jiangxi Health-Biotech Stem Cell Technology Co., Ltd., Shangrao, 334000 China; 6 Key Laboratory of Molecular Diagnostics and Precision Medicine for Surgical Oncology in Gansu Province, Gansu Provincial Hospital, 204 Donggangxi Road, Chengguan District, Lanzhou City, 730013 Gansu Province China; 7 State Key Laboratory of Experimental Hematology & National Clinical Research Center for Blood Disease, Institute of Hematology & Blood Diseases Hospital, Chinese Academy of Medical Sciences & Peking Union Medical College, Tianjin, 300020 China; 8 Stem Cell Bank of Guizhou Province, Guizhou Health-Biotech Biotechnology Co., Ltd., Guiyang, 550000 China

## Abstract

Natural killer (NK) cells are unique innate immune cells and manifest rapid and potent cytotoxicity for cancer immunotherapy and pathogen removal without the requirement of prior sensitization or recognition of peptide antigens. Distinguish from the T lymphocyte-based cythotherapy with toxic side effects, chimeric antigen receptor-transduced NK (CAR-NK) cells are adequate to simultaneously improve efficacy and control adverse effects including acute cytokine release syndrome (CRS), neurotoxicity and graft-versus-host disease (GVHD). Moreover, considering the inherent properties of NK cells, the CAR-NK cells are “off-the-shelf” product satisfying the clinical demand for large-scale manufacture for cancer immunotherapy attribute to the cytotoxic effect via both NK cell receptor-dependent and CAR-dependent signaling cascades. In this review, we mainly focus on the latest updates of CAR-NK cell-based tactics, together with the opportunities and challenges for cancer immunotherapies, which represent the paradigm for boosting the immune system to enhance antitumor responses and ultimately eliminate malignancies. Collectively, we summarize and highlight the auspicious improvement in CAR-NK cells and will benefit the large-scale preclinical and clinical investigations in adoptive immunotherapy.

## Introduction

Anticancer immunotherapies, including adoptive cytotherapy and checkpoint inhibitors, have present as novel pillars with oncology management [1, 2]. For decades, pioneering investigators have devoted to verify the interactions between the responses of the human immune system and numerous cancers or invaders such as bacteria and viruses, which collectively accelerate the development of clinically effective cancer immunotherapy [3, 4]. However, those reported “immune enhancement” strategies have a series of disadvantages such as rare objective responses and concomitant immune-related adverse events (irAEs), which could be largely alleviated by the termed “immune normalization” (e.g., PD-1/PD-L1) with more beneficial cancer response-to-toxicity profile [4].

Current studies have indicated the diversity of cancer immunotherapies together with the potentially combined strategies in multiple indications [5, 6]. For instance, we and other investigators have reported the successful generation of T lymphocyte-mediated tactics including chimeric antigen receptor-modified T (CAR-T) cells and T cell receptor-engineered T (TCR-T) cells as well as tumor-infiltrating lymphocytes (TILs) and regulatory T (Treg) cells in eliminating malignant hematologic tumors (e.g., B acute lymphoblastic leukemia) and metastatic solid tumors (e.g., HBV-related hepatocellular carcinoma, head and neck squamous cell carcinoma) processed by antigen-presenting cells (APCs) and fine-tuned by co-stimulatory or co-inhibitory signals [6–12]. However, the adoptive T cell-based immunotherapy severely constrained by the major limitation of the rapidly declined cellular viability and function, together with the requirement of concurrent administration of the adjuvant drugs after transplantation [13]. Moreover, due to the alteration in genetic mutation and cell-surface biomarker expression and the resultant off-target effects, tumor escape has become a common but intractable outcome of malignant transformation and identification of more optimal candidates or personalized neoantigens seems boundless [14–16]. Collectively, the autologous T lymphocytes, including the classical T cell receptor-engineered T (TCR-T) cells and CAR-transduced T (CAR-T) cells, are labor-intensive to manufacture and logistically challenging to personalized deliver to inpatients.

In consequence, state-of-the-art renewal has turned to rediscover the immune recognition and eradication of tumor cells by comminating with immune checkpoint blockade (e.g., CTLA4, PD-1/PD-L1), and in particular, to harness the innate immune response with moderate cytotoxicity and reduced adverse effects [17, 18]. Of the indicated innate immune cells such as macrophages (Mø) and dendritic cells (DCs), autologous or allogeneic NK cells are adequate to fulfill the biofunction of combating malignant tumors and pathogenic microorganisms via paracrine effects (e.g., IFN-γ, GM-CSF), antibody-dependent cell-mediated cytotoxicity (ADCC) and direct cytolytic effect dispense with preliminary antigen presentation as well as manipulating other immune contextures to recognize and attack cancer cells [1, 5, 19–21]. However, the heterogeneous tumor cells with genetic or epigenetic variations are also sufficient to elude the immunological surveillance and even reversely suppress NK cell cytotoxicity by interdicting the corresponding activating receptors [5, 22, 23]. Considering the deficiency of CAR-T and non-gene-edited NK cells, CAR-NK cells have been recognized as novel therapeutic options aiming at reducing the incidence of relapse and attaining complete remission. Of note, considerable progresses have been achieved in an increasing number of therapeutic dimensions ranging from preclinical studies to clinical practices [24].

Therefore, in this review article, we mainly summarize the key elements and current advances of CAR-NK cell-based immunotherapy including cell sources, novel target selection, design of CAR construction, mode of CAR transduction, and ultimately discuss the opportunities and challenges of adoptive CAR-NK cell-based cancer immunotherapy. Collectively, the CAR-NK cell-mediated cytotherapy has constituted a promising area of cellular immunotherapy innovation.

## Cell sources for CAR-NK cells

### NK cell lines

**Fig. 1:**
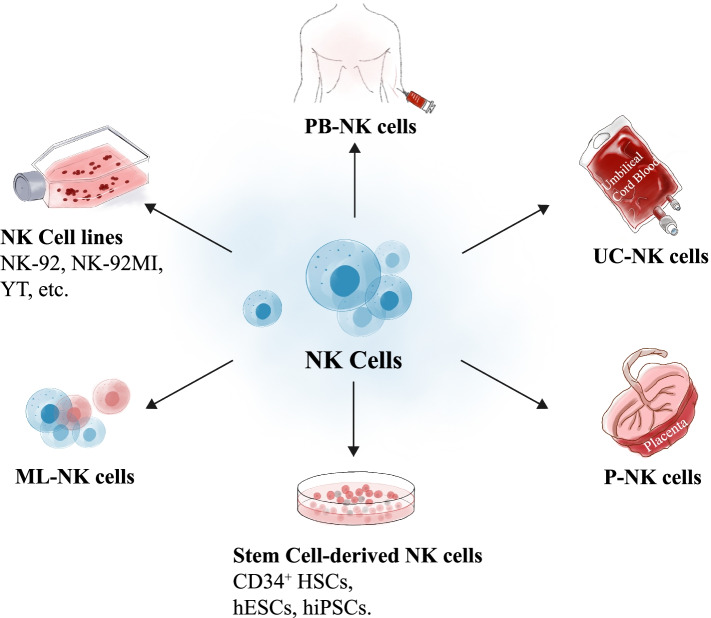
The schematic diagram of the sources of NK cells

Generally, considering the difficulty in isolating, purifying and expanding primary NK cells as well as the inefficiency in transducing CAR constructs, the well-established NK cell lines with indefinite expansion capacity have been used in clinical practice [25]. Notably, the representative IL-2 depend NK-92 cell line and the NK-92MI derivation exhibit splendid advantages of easy expansion, cultivation and activation in the context of lymphodepletion, together with sustainable and reliable cytotoxicity after infusion against leukemia cells (Fig. 1) [24]. For example, Boyiadzis and the colleagues conducted a phase 1 clinical trial of NK92 cell-based adoptive immunotherapy in patients with refractory and relapsed acute myeloid leukemia (AML) and confirmed the feasibility, safety and strong anti-leukemia activity [26]. Simultaneously, it’s noteworthy that NK92 cells are originated from patients with non-Hodgkin’s lymphoma and thus require irradiation prior to infusion to eliminate risks of malignant transformation and the accompanied chromosomal abnormalities [27]. Another preclinical study upon AML immunotherapy by Kloess et al verified that engineered CD123-CAR-NK-92 cells showed higher levels of granzyme and interleukin secretion and preferable cytotoxic activities over the primary human donor-derived CD123-CAR-NK cells, while revealed significant side effects against nonmalignant cells as well [28]. Additionally, Binyamin et al. found that NK-92 cells revealed enhanced ADCC by blocking the inhibitory receptors (e.g., KIR2DL1, KIR3DL1) and combining with rituximab [29].

Another classical NK cell line YT with the prostate cancer cell antigen PSMA transduction and shp-2 (PTPN11) deletion has been indicated with enhanced cytotoxicity [30]. Meanwhile, the leukemic cell line YT has also been proved with spontaneous cytotoxicity against B lymphoma and specifical lytic effect upon AML by targeting CD80^+^/CD86^+^ B lymphoblastoid cells and CD33^+^ leukemia cells, respectively [31, 32]. Despite the increasing references of CAR-NK cell-based cancer immunotherapy, yet most of the current studies are preclinical. However, the observations of the existing studies are favor of novel treatment concepts employing CAR-NK cell lines with potent degranulation and selective cytotoxicity in malignancies [33].

### Peripheral blood-derived NK (PB-NK) cells

In peripheral blood, NK cells account for a proportion of 5–20% of leukocytes, which are divided into the dominating CD56^dim^CD16^high^ subset (85–95%) and the minimal CD56^bright^CD16^low/neg^ subset (5–15%) [5, 34]. Besides, PB-NK cells express a wide range of active receptors and thus hold potential as splendid sources for adoptive CAR-NK cell generation (Fig. 1) [21, 35]. In general, resting PB-NK cells reveal a tremendous proliferative CD3^−^CD56^bright^ cellular phenotype and are capable of secreting immunomodulatory cytokines, while the CD3^−^CD56^dim^ counterpart possesses highly cytotoxicity and poor proliferation in response to cytokine stimulation (e.g., IL-2) [36].

To date, autogenous and allogeneic donor-derived PB-NK cells and CAR-PB-NK cells have been most effective in the treatment of acute leukemia (clinically effective doses ranging from 1 × 10^6^/kg to 9.3 × 10^6^/kg) whereas with relatively minimal activity against solid tumors [37–39]. Recently, a preclinical study by Quintarelli et al confirmed that CD19-CAR-transduced PB-NK cells were sufficient to mediate robust cytotoxicity against B-cell precursor acute lymphocytic leukemia (Bcp-ALL) and maintain the function of all “native” NK coreceptors after genetic modification [40].

### Umbilical cord blood-derived NK (UC-NK) cells

Differ from PB-NK cells, NK cells in umbilical cord blood (UC-NK) only account for a proportion of approximately 5% of total mononuclear cells (TNCs) but offer unique alloreactive advantages for adoptive immunotherapy and boost the potential as a third-party product for extensive clinical scalability (Fig. 1) [36, 41]. In spite of the higher percentage of naïve NK cell population in circulating umbilical cord blood, yet most of the UC-NK cells were adequate to differentiate into functionally mature and active effector cells and thus the successful acquisition of functional competence after ex vivo co-stimulation with cytokine cocktails (e.g., IL-2, IL-7, IL-12, IL-15, IL-18) [36, 42]. For example, Xing et al reported the low cytolytic activity of resident UC-NK cells in vivo attribute to impaired lytic immunological synapse formation as well as the enhancement by IL-2 stimulation during ex vivo expansion and activation [43]. Collectively, the existing literatures indicate that UC-NK cells are phenotypically and functionally immature but are capable of maturation [42].

Of note, an increasing number of investigators have turned to UC-NK cells for generating preclinically or clinically tested CAR-NK cells [44]. For instance, a fist-in-human phase 1/2 clinical trial identified the feasibility of lympho-depleted CAR-UC-NK cells for the treatment of recurrent and refractory CD19^+^ B-cell lymphoma including seven cases with complete remissions without causing major toxic effects [45]. Despite the once reported generation of over 100 engineered CAR-NK cell doses from one cord blood unit, yet the UC-NK cells have noteworthy limitations in cell mass for large-scale adoptive immunotherapy but with high level of the inhibitory receptor NKG2A expression and poor in vitro cytotoxicity [36, 42, 46].

### Placental blood-derived NK (P-NK) cells

In spite of the rare content of P-NK cells (< 2%) in TNCs, yet placental blood is more abundant compared to the aforementioned adult peripheral blood and umbilical cord blood (Fig. 1). Meanwhile, current strategies have indicated the high-efficient generation of clinical-grade CD3^−^CD56^+^ NK cells (an average of nearly 1.0 billion NK cells per donor) with remarkably increased antitumor cytolytic activity from placenta perfusate [38]. Compared to UC-NK cells, the derived P-NK cells are largely similar to UC-NK cells phenotypically and functionally, but display distinct microRNA expression profiles, immunophenotypes and superiority in killing a wide range of cancer cell lines in vitro and thus hold potential for CAR-P-NK cell-based immunotherapeutic development [38, 47]. Notably, Guo et al very recently raised the possibility of enhancing cytotoxicity of P-NK cells via CRISPR/Cas9-induced CBLB ablation [48].

### Stem cell-derived NK cells

Currently, prospective studies have also indicated the feasibility of deriving mature NK cells from CD34^+^ hematopoietic stem/progenitor cells (HSPCs). In a preclinical study upon AML xenograft model, Cany et al reported the proof-of-concept safety and efficiency of targeting bone marrow-residing leukemia cells via the CCR6/CCL20 and CXCR3/CXCL10–11 axis in NOD/SCID/IL2Rg^null^ mice [49].

Human pluripotent stem cells (hPSCs), including human induced pluripotent stem cells (hiPSCs) and human embryonic stem cells (hESCs), possess self-renewal and multi-lineage differentiation potential [50–52]. During the past decades, we and other investigators have reported the generation of progenitor cells and functional cells from hPSCs including mesenchymal stem/stromal cells (MSCs), megakaryocytes (MKs) and NK cells [50, 53]. Notably, differ from other cell sources with dominating limitations in NK cell survival and proliferation, hPSC-NK cells can be manufactured from the standardized hPSC population and thus satisfy the clinical demands for large-scale, homogeneous CAR-NK cell products (Fig. 1) [54, 55]. Meanwhile, considering the relatively low efficiency of CAR transduction into primary NK cells, the deficiency of adult PB-NK cells and perinatal UC-NK cells and P-NK cells in cellular activity against solid tumors might be overcome by the genetically modified CAR-hPSC-NK cells via viral or non-viral strategies as hypothesized by pioneering investigators [54, 56].

Moreover, it is considered that CARs can be easily and conveniently delivered into hPSCs by utilizing the non-viral transgenic strategy [57, 58]. For example, Ni and the colleagues reported the integration of chimeric receptor CD4ζ into hPSC-derived NK cells (CD4ζ-hESC-NK cells, CD4ζ-hiPSC-NK cells) with improved efficacy upon human immunodeficiency virus (HIV)/AIDS [57]. Of note, Li et al recently highlighted the feasibility of transducing CAR constructs with conventional NK cell-specific intracellular activating domains into iPSC-NK cells (CAR^+^ iPSC-NK cells) for the further optimization of tumor-specific recognition and cytotoxicity [59].

### Memory-like NK (ML-NK) cells

Cytokine-induced ML-NK cells with the dominant NKG2A checkpoint expression, phenotypically distinct from the in vivo conventional NK cells, have been considered safe and sufficient to induce remissions in patients with AML, which are recognized as new avenues to facilitate CAR-NK cell therapeutics (Fig. 1) [60, 61]. Therefore, we and other investigators presume that CAR-ML-NK cells possess more potent and flexible response to a variety of cancer cell-associated triggers compared to the conventional NK cells [62, 63]. It’s noteworthy that the differentiated memory-like CAR-NK cells displayed elevated activating receptors against myeloid leukemia and prolonged survival in vivo dispense with the typical KIR-KIR ligand interactions [61, 64]. Meanwhile, numerous preclinical data have demonstrated the superior degranulation and IFN-γ-associated response of CAR-NK cells as well as enhanced cancer cell killing and ADCC effect against tumor cells [65, 66].

Notably, peripheral blood-derived ML-NKs with a truncated CD-19-CAR transduction were phenotypically and functionally mature and manifested significantly raised IFN-γ secretion and degranulation, broaden recognition and specific killing against NK-resistant lymphoma compared to the conventional CAR-NK cells or no-specific NK cells [63]. Moreover, Foltz et al reported that the CAR-NK cells could even survive and persist in vivo for over 2 months following adoptive transfer in the immune compatible environment, which would be significant improvement of the short lifespan of conventional NK cells.

## Targets for CAR-NK cells

**Fig. 2:**
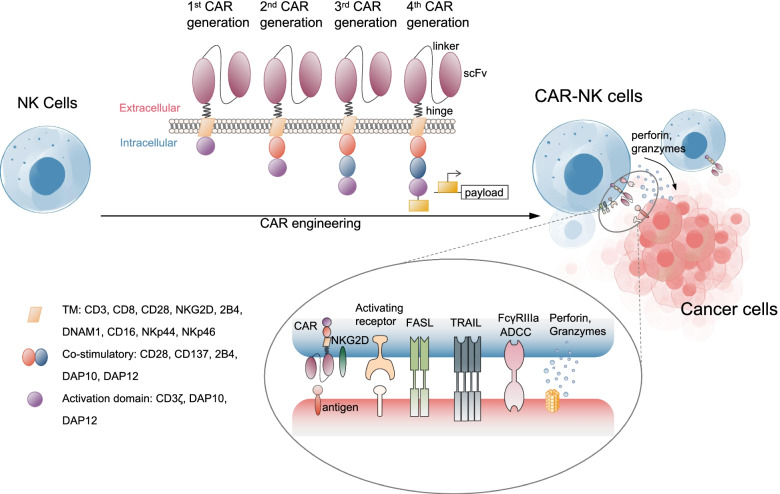
The overview of the constructs and targets of CAR-NK cells

Aiming to generate novel CAR-NK cell-based cancer therapeutics, the consideration of tumor-specific surface antigens and the costimulatory molecules is the first-line decision to be made by investigators [55]. Direct transfer of CAR structures involved in CAR-T (e.g., CD19, CD3ζ, 4-1BB, CD28) into NK cells is the predominantly initial CAR-NK cell-based studies (Fig. 2, Table 1) [111, 112]. For instance, there are a series of activating receptors such as TNF-related apoptosis-inducing ligands (TRAILs), co-stimulatory receptors (e.g., CD244, CD137) and the well-established subsets (e.g., FcgRIIIa, FasL, NKG2D, NKp44, NKp46), which are capable of provoking cytolytic programs via intra-cytoplasmic ITAMs (e.g., 2B4, 41BB) [59, 113, 114]. Significant efforts have been made to enhance CAR-NK cell responses against surface antigens by multiple targeted activation such as CD19, CD20, CD22, CD276, CD138, CS1, HER-2, NKG2D and GD 2 [5, 67, 69, 115, 116].

**Table 1:** Targets for CAR-NK cell generation

Target	Tumor	References
CD19	Acute lymphocytic leukemia (ALL)	Romanski, et al [67]
CD7	Acute lymphocytic leukemia (ALL)	You, et al [68]
CD5	Acute lymphocytic leukemia (ALL)	Xu, et al [69]
FLT3	Acute lymphocytic leukemia (ALL)	Oelsner, et al [70]
CD33	Acute myelocytic leukemia (AML)	Tang, et al [71]
CD123	Acute myelocytic leukemia (AML)	Klöß, et al [72]
CD4	Acute myelocytic leukemia (AML)	Pinz, et al [73]
HER2	Breast cancer	Schönfeld, et al [74]
EpCAM	Breast cancer	Sahm, et al [75]
TF	Breast cancer	Hu, et al [76]
EGFR	Breast cancer	Chen, et al [77]
NKG2D	Breast cancer	Chang, et al [78]
CD19	Chronic lymphocytic leukemia (CLL)	Boissel, et al [79]
EpCAM	Colorectal cancer	Zhang, et al [80]
CEA	Colorectal cancer	Shiozawa, et al [81]
NKG2D	Colorectal cancer	Xiao, et al [82]
GD2	Ewing sarcoma	Kailayangiri, et al [83]
HER2	Gastric cancer	Wu, et al [84]
EGFRvIII	Glioblastoma	Han, et al [85]
EGFR	Glioblastoma	Han, et al [85]
CD73	Glioblastoma	Wang, et al [86]
HER2	Glioblastoma	Zhang, et al [87]
ROBO1	Glioma and Neuroblastoma	Qu, et al [88]
GPC3	Hepatocellular cancer (HCC)	Huang, et al [89]
NKG2D	Hepatocellular cancer (HCC)	Chang, et al [78]
c-MET	liver cancer	Liu, et al [90]
NKG2D	Lung cancer	Lu, et al [91]
**Target**	**Tumor**	**References**
B7-H3	Lung cancer	Yang, et al [92]
CD19	Lymphoma	Gang, et al [63]
CD4	Lymphoma	Pinz, et al [73]
GPA7	Melanoma	Zhang, et al [93]
CD138	Multiple Myeloma	Jiang, et al [94]
CS1	Multiple Myeloma	Chu, et al [95]
BCMA	Multiple Myeloma	Ng, et al [96]
GD2	Neuroblastoma	Esser, et al [97]
CD276	Neuroblastoma	Elahi, et al [98]
aFR	Ovarian cancer	Ao, et al [99]
HER2	Ovarian cancer	Kruschinski, et al [100]
Mesothelin	Ovarian cancer	Cao, et al [101]
GPC3	Ovarian cancer	Ueda, et al [102]
NKG2D	Ovarian cancer	Ng, et al [103]
Mesothelin	Pancreatic cancer	Batchu, et al [104]
ROBO1	Pancreatic cancer	Li, et al [105]
PSMA	Prostate	Montagner, et al [106]
HER2	Renal cell carcinoma (RCC)	Schonfeld, et al [74]
EGFR	Renal cell carcinoma (RCC)	Zhang, et al [107]
PSCA	Ladder carcinoma	Topfer, et al [108]
HLA-G	Kidney renal clear cell carcinoma, Kidney renal papillary cell carcinoma, Pancreatic ductal adenocarcinoma, Thyroid cancer	Jian, et al [109]
Mesothelin	Ovarian cancer	Li, et al [59]
CD20	Lymphoma, Leukemia cells	Muller, et al [110]
NKG2D	Osteosarcoma	Chang, et al [78]

Nowadays, several groups further indicated the re-designment of CAR-NK cells with NK cell signaling-associated domains to enhance the antitumor efficacy by improving cytotoxicity and INF-γ secretion such as DAP-10, DAP-12, 2B 4 [117]. Nevertheless, due to the rare tumor-specific cell-surface antigens, CAR-NK cells also endure the main disadvantages of requiring extracellular surface expression of specific targets on cancer cells, which thus restrict the broadness and specificity of CAR-NK cell application [4, 118]. To overcome the short lifespan and transient cytotoxic activity of CAR-NK cells, Zhang et al recently transduced the homodimers and heterodimers of ErbB2/HER2-specific CARs with CD3ζ and composite CD28ζ signaling domains into NK-92/63.z cells to induce long-lasting endogenous cytotoxicity against immunocompetent glioblastoma (Fig. 2, Table 1) [87].

## Construction of CARs

Generally, the CARs are engineered receptor proteins to enable NK cells with novel ability to target cancer cell-specific antigen proteins, which are composed of an intracellular activating signaling domain, a transmembrane region and an extracellular antigen binding domain (Fig. 2, Table 1) [55]. The intracellular activating signaling domains (e.g., CD137, CD28) mainly mediate the activation and cytotoxicity of CAR-NK cells, while the extracellular antigen binding domains (e.g., the single-chain variable fragments) recognize the specific antigen of tumor cells [21]. For example, Quintarelli and the colleagues reported the successful design of retroviral plasmid carrying the cassettes of a second-generation CD19-CAR construct and transduced into PB-NK cells for Bcp-ALL management [40]. Simultaneously, another preclinical study reported the splendid efficiency of CAR-NK-92 and CAR-NK-92MI cells with the second- or third-generation CARs targeting CD3ζ and CD5 domains against mouse model of T-cell malignancies, respectively [69, 119]. Very recently, Daher et al took advantage of the fourth-generation “armored” CARs for generating CAR-UC-NK cells by targeting the cytokine-inducible SH2-containing (CIS) protein, which efficiently boosted NK cell antitumor activity against lymphoma xenografts and resulted in enhanced aerobic glycolysis [41, 120].

Therewith, due to the potentially excessive cytokine secretion of CAR-NKs, there is a possibility that the fourth-generation of CARs might cause unanticipated toxicity and should reinsert suicide genes (Fig. 2, Table 1) [121]. Collectively, the genetically engineered CAR-NK cells contain a typically extracellular antigen-binding domain, a hinge and transmembrane region and the concomitant intracellular costimulatory domains from receptors, which represents the paradigmatic design of utilizing engineered NK cells for effective attack of cancer cells [4, 122]. Of note, the choice and design of the NK cell activating receptor (e.g., NKG2D, DAP10) and remaining domains of the CAR construct, including the aforementioned transmembrane domains (e.g., CD28), co-stimulatory domains and signaling domains (e.g., CD3ζ), together with the NK cell subtypes, should be taken into incorporated consideration [55].

## CAR transduction

CAR-transduced cytotherapy was initially advocated using autologous T lymphocytes (CAR-T) and obtained great success in treating hematological malignancies whereas marginal success in facing solid tumors largely attributes to the highly immunosuppressive cancer microenvironment [9, 122, 123]. Advances in genome editing technique and the applicability of the approach have vastly accelerated the development of designer CAR-NK cell therapy products, which are currently being tested in both preclinical studies and clinical trials [55].

### Retrovirus

For decades, a plethora of groups have reported the successful transduction of CAR constructs into expanded NK cells by utilizing a single round of retroviral vector-based method ranging from 27 to 52%, and in particular, an even higher efficient transduction (approximately 70%) has been achieved by Imamura and the colleagues for IL-15 expression [124, 125]. Strikingly, Daher and the colleagues compared the retrovirus transfection of iC9.CAR19.CD28-z-2A-IL-15 (with IL-15) with CAR19.CD28-z (without IL-15) into UC-NK cells, and confirmed the feasibility of high-efficient CAR-NK cell generation and the persistence of in vivo antitumor activity in xenogeneic lymphoma models [41]. Furthermore, Suerth et al systematically compared different retroviral pseudotypes, retroviral vector systems and transduction protocols, and verified that the highest (up to 60%) transduction levels of CAR-19 expression cassette were achieved with α-retroviral plasmids into primary human NK cells [126]. Simultaneously, the retrovirus-based gene-delivery vehicles also have main obstacles to widespread clinical applications including the immunogenicity and insertional mutagenesis as well [58].

### Lentivirus

As with retrovirus, lentiviral also have been widely used by preclinical studies for CAR vector transduction into NK cells such as UC-NK cells, PB-NK cells, iPSC-NK cells and ML-NK cells. For example, talented investigators utilized the scFvs-based CD33-CAR and CD19-CAR lacking the immunoreceptor tyrosine-based activation motif (ITAM) domains to manufacture the second-generation CARs with CD3ζ and CD137 intracellular domains [63, 127, 128]. Simultaneously, Oelsner and other investigators verified that gene-modified CAR-NK-92 cells after lentiviral-mediated gene transfer displayed stable and homogeneous CD-19-and CD20-specific CAR expression, high and specific ADCC against lymphoma and leukemia cells [5, 33, 129].

### Nonviral-mediated transfection

To overcome the major barriers of low-efficient exogenous gene transfer into the primary NK cells, a certain number of research groups turn to the nonviral-mediated transfection such as lipofection and electroporation [58, 130]. Of them, the nonviral plasmids are emerging as promising alternatives and facilitate clinical CAR-NK cell-based cancer immunotherapy [58]. Compared to the aforementioned virus-mediated strategies, the nonviral methods revealed transient and rapid expression of CAR-conjunct genes as well as less variability and apoptosis, whereas the generated CAR-NK cells showed declined expression level of transgenes due to the non-integrated property [58]. Of note, the current progresses of transfection technologies, including the DNA transposon systems composed of the PiggyBac (PB) and the sleeping beauty (SB) subsets, are sufficient to deliver CAR structures into the genomes of primary NK cells or iPSCs with long-lasting transgene expression [131].

Collectively, the transposon systems for high-efficient CAR-NK cell generation have splendid advantages such as increased biosafety, rapid and persistent transgene expression, low immunogenicity, cost-effect and capacity for large gene fragment (> 100 kb) transduction, which make them attractive options for CAR-NK cell manufacturing [130]. Nevertheless, considering the dissatisfactory efficiency in current transduction and the potential advances in CAR construct design, the next-generation of CAR-NK cells might cause fewer cytotoxicity and even ultimately eliminate the demands for suicide genes or safety switches [121].

## CAR-NKs in cancer immunotherapy

**Fig. 3:**
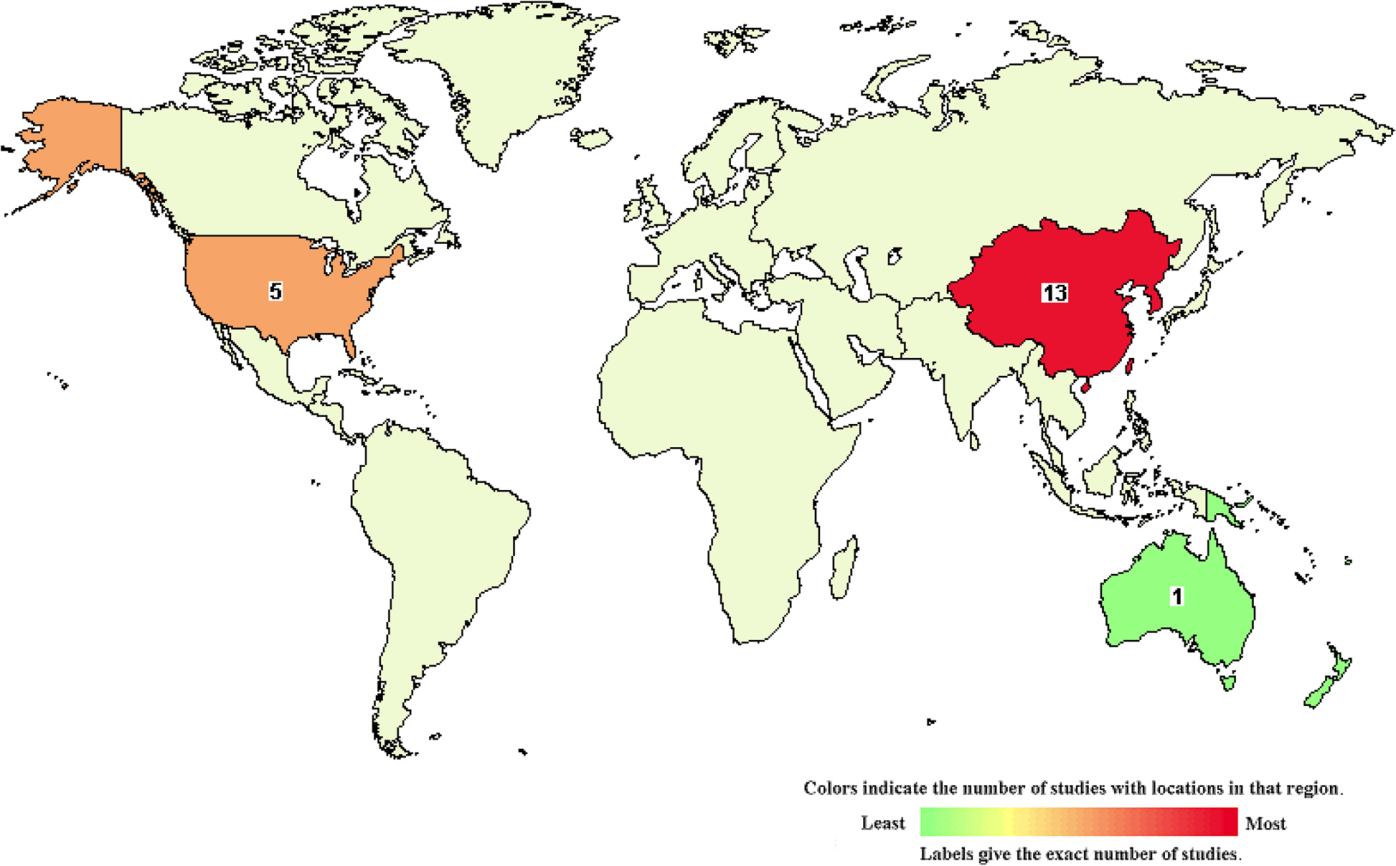
The distribution of CAR-NK cell-based clinical trials worldwide

Nowadays, CAR-NK cell-mediated immunotherapy has grown exponentially and emerged as an alternative treatment option for patients with metastatic malignancies (Fig. 3, Table 2). Despite the manifestation of CAR-NK cells in cancer immunotherapy has been extensively explored, yet most of the applications in numerous tumor models are relatively restricted and mainly staying at the preclinical stage [5, 24]. Clinically approved second-generation CAR-NK cells usually contain the CD3ζ domain in combination with either a 4-1BB (Kymriah®) or CD28 (Yescarta®) co-stimulatory domain, and prominently focus on CD19^+^ lymphoid-derived hematologic malignancies [55].

**Table 2:** Clinical trials of CAR-NK cell-based cancer immunotherapy

Rank	Status	Study Title	Conditions	Interventions	Phase	Number Enrolled	NCT Number	Study Complete	Location
1	Recruiting	Clinical Research of ROBO1 Specific CAR-NK Cells on Patients with Solid Tumors	Solid Tumor	Biological: ROBO1 CAR-NK cells	Phase 1, Phase 2	20	NCT03940820	May-2022	China
2	Unknown	Pilot Study of NKG2D-Ligand Targeted CAR-NK Cells in Patients with Metastatic Solid Tumours	Solid Tumor	Biological: CAR-NK cells targeting NKG2D ligands	Phase 1	30	NCT03415100	Dec-2019	China
3	Recruiting	Clinical Research of ROBO1 Specific BiCAR-NK/T Cells on Patients with Malignant Tumor	Malignant Tumor	Biological: BiCAR-NK/T cells (ROBO1 CAR-NK/T cells)	Phase 1, Phase 2	20	NCT03931720	May-2022	China
4	Not yet recruiting	Study of Anti-PSMA CAR NK Cell in Castration-Resistant Prostate Cancer	Castration-resistant Prostate Cancer	Biological: anti-PSMA CAR NK cells	Early Phase 1	9	NCT03692663	Dec-2021	
5	Not yet recruiting	Immunotherapy Combination: Irradiated PD-L1 CAR-NK Cells Plus Pembrolizumab Plus N-803 for Subjects with Recurrent/ Metastatic Gastric or Head and Neck Cancer	Gastroesophageal Junction (GEJ) Cancers; Advanced HNSCC	Drug: N-803; Drug: Pembrolizumab; Biological: PD-L1t-haNK	Phase 2	55	NCT04847466	Dec-2025	United States
6	Not yet recruiting	Study of Anti-Mesothelin Car NK Cells in Epithelial Ovarian Cancer	Epithelial Ovarian Cancer	Biological: anti-Mesothelin Car-NK cells	Early Phase 1	30	NCT03692637	Nov-2021	
7	Recruiting	Clinical Research of ROBO1 Specific BiCAR-NK Cells on Patients with Pancreatic Cancer	Pancreatic Cancer	Biological: BiCAR-NK cells (ROBO1 CAR-NK cells)	Phase 1, Phase 2	9	NCT03941457	May-2022	China
8	Recruiting	NKX019, Intravenous Allogeneic Chimeric Antigen Receptor Natural Killer Cells (CAR NK), in Adults With B-cell Cancers	Lymphoma, Non-Hodgkin; B-cell Acute Lymphoblastic Leukemia; Large B-cell Lymphoma	Biological: NKX019	Phase 1	60	NCT05020678	Jul-2038	United States; Australia
9	Recruiting	Cord Blood Derived Anti-CD19 CAR-Engineered NK Cells for B Lymphoid Malignancies	Acute Lymphocytic Leukemia; Chronic Lymphocytic Leukemia; Non-Hodgkin’s Lymphoma	Drug: Fludarabine; Cyclophosphamide; CAR-NK-CD19 cells	Phase 1	27	NCT04796675	Mar-2024	China
10	Recruiting	Umbilical & Cord Blood (CB) Derived CAR-Engineered NK Cells for B Lymphoid Malignancies	B-Lymphoid Malignancies; Acute Lymphocytic Leukemia; Chronic Lymphocytic Leukemia; Non-Hodgkin Lymphoma	Drug: Fludarabine; Drug: Cyclophosphamide; Drug: Mesna;	Phase 1, Phase 2	36	NCT03056339	Jun-2022	United States
11	Recruiting	Universal Chimeric Antigen Receptor-modified AT19 Cells for CD19 + Relapsed/Refractory Hematological Malignancies	Acute Lymphoblastic Leukemia; Chronic Lymphoblastic Leukemia; B-cell Lymphoma	Drug: Fludarabine; Cyclophosphamide; CAR-NK-CD19 cells	Phase 1	27	NCT04796688	Mar-2024	China
**Rank**	**Status**	**Study Title**	**Conditions**	**Interventions**	**Phase**	**Number Enrolled**	**NCT Number**	**Study Complete**	**Location**
12	Not yet recruiting	Anti-BCMA CAR-NK Cell Therapy for the Relapsed or Refractory Multiple Myeloma	Multiple Myeloma, Refractory	Biological: Anti-BCMA CAR-NK Cells; Drug: Fludarabine; Drug: Cytoxan	Early Phase 1	27	NCT05008536	Sep-2023	China
13	Recruiting	NKX101, Intravenous Allogeneic Engineered Natural Killer Cells, in Adults with AML or MDS	Relapsed/Refractory AML; AML, Adult MDS; Refractory Myelodysplastic Syndromes	Biological: NKX101 - CAR NK cell therapy	Phase 1	64	NCT04623944	Jul-2038	United States;
14	Not yet recruiting	Anti-CD33 CAR NK Cells in the Treatment of Relapsed/Refractory Acute Myeloid Leukemia	Leukemia, Myeloid, Acute	Biological: anti-CD33 CAR NK cells; Drug: Fludarabine; Drug: Cytoxan	Phase 1	27	NCT05008575	Sep-2023	China
15	Unknown	CAR-pNK Cell Immunotherapy for Relapsed/Refractory CD33+ AML	Acute Myelogenous Leukemia; Acute Myeloid Leukemia; Acute Myeloid Leukemia with Maturation	Biological: anti-CD33 CAR-NK cells	Phase 1, Phase 2	10	NCT02944162	Sep-2018	China
16	Recruiting	Clinical Research of Adoptive BCMA CAR-NK Cells on Relapse/Refractory MM	Multiple Myeloma	Biological: BCMA CAR-NK 92 cells	Phase 1, Phase 2	20	NCT03940833	May-2022	China
17	Not yet recruiting	Study of Anti-CD22 CAR NK Cells in Relapsed and Refractory B Cell Lymphoma	Refractory B-Cell Lymphoma	Biological: Anti-CD22 CAR NK Cells	Early Phase 1	9	NCT03692767	Nov-2021	
18	Not yet recruiting	Study of Anti-CD19 CAR NK Cells in Relapsed and Refractory B Cell Lymphoma	Refractory B-Cell Lymphoma	Biological: Anti-CD19 CAR NK Cells	Early Phase 1	9	NCT03690310	Nov-2021	
19	Not yet recruiting	Anti-CD19 CAR NK Cell Therapy for R/R Non-Hodgkin Lymphoma.	NHL	Biological: anti-CD19 CAR NK	Early Phase 1	9	NCT04639739	Dec-2023	China
20	Recruiting	Clinical Study of HLA Haploidentical CAR-NK Cells Targeting CD19 in the Treatment of Refractory/Relapsed B-cell NHL	B-cell Non-Hodgkin Lymphoma	Biological: anti-CD19 CAR-NK	Phase 1	25	NCT04887012	May-2024	China
21	Unknown	Study of Anti-CD19/CD22 CAR NK Cells in Relapsed and Refractory B Cell Lymphoma	Refractory B-Cell Lymphoma	Biological: Anti-CD19/CD22 CAR NK Cells	Early Phase 1	10	NCT03824964	Jan-2021	
22	Unknown	PCAR-119 Bridge Immunotherapy Prior to Stem Cell Transplant in Treating Patients with CD19 Positive Leukemia and Lymphoma	Acute Lymphocytic Leukemia; Chronic Lymphocytic Leukemia; Follicular Lymphoma	Biological: anti-CD19 CAR-NK cells	Phase 1, Phase 2	10	NCT02892695	Sep-2019	China
23	Withdrawn	CAR.CD19-CD28-zeta-2A-iCasp9-IL15-Transduced Cord Blood NK Cells, High-Dose Chemotherapy, and Stem Cell Transplant in Treating Participants With B-cell Lymphoma	CD19 Positive Mantle Cell Lymphoma; Recurrent Diffuse Large B-Cell Lymphoma	Procedure: Autologous Hematopoietic Stem Cell Transplantation; Drug: Carmustine; Drug: Cytarabine	Phase 1, Phase 2	0	NCT03579927	Oct-2019	United States

### Hematological malignancies

**Fig. 4:**
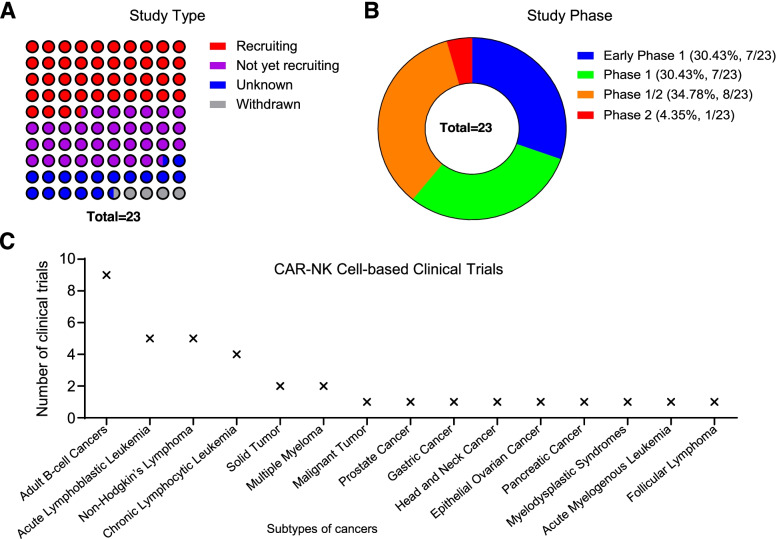
The dissection of CAR-NK cell-based clinical trials A-B. The status (**A**) and phase (**B**) of CAR-NK cell-based clinical trials. **C**. The detailed distribution of CAR-NK cell-based clinical trials in cancer immunotherapy

Similar to CAR-T-based immunotherapy, CAR-NK cells were initially introduced to combat with hematological malignancies such as lymphoma, myeloma and leukemia (Fig. 4, Table 2) [121, 122]. Current studies have indicated the preferable efficiency of CD19-CAR-NK cells in combating lymphoid malignancies over CAR-T-based cellular immunotherapy, which largely attribute to the aforementioned merits [55, 132]. For example, a preclinical study took advantage of the UC-NK cells and the fourth-generation iCas9.CAR-19-CD28-ζ-IL15 plasmid with a suicide gene for Raji lymphoma xenograft model treatment, the CD19-CAR-UC-NK cells exhibited preferable antitumor efficacy and enhanced persistence over the non-transduced UC-NK cells [46].

Simultaneously, the first large-scale trial of HLA-mismatched CAR-UC-NK cell-based immunotherapy (ClinicalTrials.gov number: NCT03056339) in combination with lymphodepleting chemotherapy for 11 patients with CD19^+^ CLL and B cell lymphoid tumors has shown safety and inspiring clinical outcomes (response rate: 73%; complete remission rate: 64%) within 30 days [45]. However, as commented by Karadimitris, CAR-UC-NK cells could also result in high rate of non-durable clinical response, which suggested the favorable initial toxicity and efficacy but uncertain durability of clinical manifestation of CAR-NK-based leukemia immunotherapy [133].

### Metastatic solid tumors

In recent years, CAR-T cell immunotherapy has revealed promising therapeutic manifestation in a series of hematologic malignancies, yet also with considerable drawbacks and limitations in metastatic solid tumor management as well (Fig. 4, Table 2) [55, 134]. Considering the intrinsic and advantaged properties, including substantially cytolytic ability, non-MHC-restricted recognition, natural infiltration in tumor tissues and convenience for their preparation as well as minimal untoward effects (e.g., CRS, GvHD and neurotoxicity), engineered CAR-NK cells are supposed as promising therapeutic option for solid tumor administration in clinical practice [54, 134, 135]. Currently, CAR-NK cells have been preclinically tested in multiple solid tumors including breast cancer, ovarian cancer, pancreatic cancer, colon cancer, glioblastoma, hepatocellular carcinoma, head and neck squamous cell carcinoma (HNSCC) [90, 117, 136, 137]. For example, numerous preclinical studies have confirmed the efficacy of CAR-NK cells upon CXCL12/SDF-1α secreting glioblastoma and epithelial cell adhesion molecule (EpCAM) positive colorectal cancer cells in xenograft model by targeting EGFRvIII via intravenous infusion [80, 138].

However, there are very limited clinical data existing on the potential of CAR-NK cells in solid tumor treatment (Fig. 4, Table 2) [117]. For instance, the outcomes of three phase I/II clinical studies of allogeneic ROBO-1-CAR-NK-92 cell-based cellular immunotherapy in pancreatic ductal adenocarcinoma (PDAC) and relative solid tumors with ROBO-1 expression for enrolled patients in China (NCT03940820, NCT03941457, NCT03931720) ulteriorly indicated the feasibility for non-hematological neoplasm treatment with CAR-NK cells [117, 135, 139]. Another phase II trial of PD-L1-CAR-NK cell-based immunotherapy combined with IL-15 agonist (N-803) and pembrolizumab (NCT04847466) is currently launched in the United States. Therefore, integration of the CAR-NK cells and antitumor drugs is promising for further altering immunosuppressive tumor microenvironment (TME) and administrating the aggressiveness and the metastatic ability of the resistant tumors [117].

## Conclusion and perspective

The heterogeneous NK cell population is advantaged immune cells with powerful cytotoxic activity and plays a unique role in both innate and adoptive immune responses, while the signatures could be eluded by tumor microenvironment [118, 140–142]. NK cells engineered with CAR expression (CAR-NK cells) have been celebrated as a landmark breakthrough of anti-tumor immunotherapy by discerning germline-encoded cell surface receptors between healthy and cancer tissues [143, 144]. Differ from NK cells and CAR-T cells, the genetically modified NK (CAR-NK) cells have superiority in further augmenting the specificity and cytotoxicity of adoptive NK cells without causing adverse effects including GvHD, CRS or immune cell- associated neurotoxicity syndrome (ICANS) [5, 60, 143, 145]. Thus, CAR-NK cells with favorable cytotoxicity, short lifespan and low manufacturing costs have been considered as promising alternatives to engineered CAR-T cells [143].

Despite the encouraging progress of CAR-NK cell-based immunotherapy, the discontented response-to-toxicity ratio and the insufficiently broad spectrum of indications further limit the application of these therapies [4]. Moreover, compared to the updates of CAR-T-based immunotherapy, the number of clinical trials and the detailed information of CAR-NK cell infusion in patients, including the in vivo spatio-temporal metabolism and host factors in the microenvironment that reversely contribute to CAR-NK cells, have not been extensively investigated [60]. Generally, compared to CAR-T cells, CAR-NK cell-based adoptive immunotherapy is also restricted to the major limitations before large-scale practical application such as insufficient capacity in proliferation and activation in vivo and durability, together with the obstacle in preparation of CAR-NK cells (e.g., low genetic transfection efficiency, low proportion of NK cells in blood, and limited amplification efficiency) [133, 146]. Conversely, CAR-NK cells have various merits over CAR-T cells due to their unique biological characteristics [117]. On the one hand, CAR-NK cells can be conveniently prepared from a wider range of autogenous and allogeneic sources without causing severe adverse reaction (e.g., aGVHD, CRS) by CAR-T-based implantation. On the other hand, CAR-NK cells are adequate for cell immunosurveillance dispense with pre-sensitization and thus have more flexible killing capacity upon both hematologic and solid tumor cells over CAR-T cells via both the CAR-dependent manner and CAR-independent intrinsic mechanisms, which thus provide alternative strategies for conquering tumor escape and varied adverse events (e.g., “on-target, off-tumor toxicity” during CAR-T application) [5, 112, 117, 147].

Nevertheless, the success of CAR-NK cells in the administration of multiple malignancies provides proof-of-principle for harnessing the immune system therapeutically, yet the short lifespan (2 weeks) and in vivo kinetics (e.g., proliferation rate, ageing) of CAR-NKs also narrows the therapeutic window and the resultant short duration of responses after infusion [122, 148, 149]. Therefore, the ex vivo expansion and activation of primary NK cells as well as the storage and shipping of NK cells for large-scale CAR-NK cell generation are prerequisites for ensuring the safety and effectiveness in vivo. For instance, a series of methodologies have been continuously developed for the persistence and activation of primary NK cells such as feeder cell stimulation (e.g., the irradiated PBMCs, K562-mb15-41BBL cells, EBV-LCLs) [150–153], cytokine cocktail (e.g., IL-2, IL15, 1 L-18) [14, 35] and physicochemical irritation (e.g., bioreactors with an assorted bag) [154]. Of note, it is great important to improve the freezing and shipping conditions (e.g., refrigeration, liquid nitrogen, drikold) for sustaining primary NK cell vitality, which will finally help CAR-NK cells necessitate adaptation of cancer immunotherapy under GMP conditions [5, 155].

Therefore, before large-scale application in cancer immunotherapy, a cohort of central issues in basic research and clinical practice of CAR-NK cells need to be improved. Firstly, many healthy tissues of the body also express cancer-associated surface antigens on tumor cells, which might cause potential off tumor or on target toxicity of CAR-NK cells. Current pioneering studies have suggested the engineering solution by inserting suicide genes or transducing genes encoding cytokines into the CAR-transduced effectors to prolong the in vivo persistence, respectively [45, 118, 156]. Secondly, the lessons learned from modified T cells (e.g., CAR-T, TCR-T) and NK cells in the administration of malignancies are worthy to be further exploited in CAR-NK cell-mediated cancer immunotherapy in future such as the optimization of large-scale NK cell-expansion approaches, antigen targets including the chimeric co-stimulatory converting receptors (CCCRs), structures with nanobodies and delivery efficiency as well as the selection of ideal patient populations in clinical trials [91, 111, 117]. Likewise, the potential tumor escape from CAR-NK cell cytotoxicity by shedding pivotal ligands and even via immunosuppressive tumor microenvironment should also take into consideration [14, 140, 157]. In particular, the pivotal signaling pathways and inhibitory checkpoints (e.g., CIS) that orchestrate CAR-NK cell “fitness” (e.g., effector function, survival and differentiation) and biofunction in tumor microenvironments have not been well defined [41, 120, 158]. Thirdly, despite CAR-NK cells are considered as “off-the-shelf” cancer immunotherapy, there’s still a long way before the large-scale generation of clinical-grade products under good manufacturing practice (GMP) and convenient to universally save the lives of inpatients with standard supervision. For example, Gaddy and Broxmeyer reported the functional maturation subset (adult-like NK activity) and the phenotypic maturation subset (adult-like CD3^−^CD56^+^CD16^+^ or CD3^−^CD56^+^CD16^−^ phenotype) of UC-NK cells by IL-2, IL-12 or IL-15 stimulation [42]. As to the recommended parameters of cell products, the manufactured CAR-NK cells should contain mostly CD3^−^CD56^+^ cells (≥90%), minimally CD3^+^ (≤0.2%) and CD14^+^ (≤5%) cells, together with the removal of endotoxin, mycoplasma and bacterial contaminations as well as contamination of co-cultured cells (≤1%) [159, 160]. Fourthly, the schedule of CAR-NK cell-based cancer immunotherapy, including the dosage, duration, kinetics and the concurrent interactions with endogenous immune cells as well as the underlying mechanisms of CAR-NK cell function, is awaiting to be optimized according to large-scale clinical trials [5, 161]. For instance, the molecular understanding of the biofunction of the natural cytotoxicity receptors (NCRs) (e.g., NKp30, NKp44, NKp46) and multidimensional immune correlations (e.g., NKG2A^+^, CD8α^+^) in cancer immunosurveillance, and in particular, the ontogenic development and maturational signals of NK cells is instrumental to exploring novel access points to combat malignancies [42, 60, 144, 162].

Moreover, the comprehensive treatment composed of a plethora of methods, including traditional therapy (e.g., surgery, radiotherapy, chemotherapy), non-cellular immunotherapy (e.g., CTLA-4, mRNA vaccine) and cellular immunotherapy (e.g., CAR-T, TCR-T, γδT, ML-NK cells) as well as auxiliary methods (TriKEs, ROCK engagers, TriNKETs) and immune-checkpoint inhibitors (e.g., CTLA-4, PD-1/PD-L1), are under clinical investigation to augment the longevity and cytotoxicity of CAR-NK cells [146, 163]. For example, a latest study upon combined approach suggested that antitumor activity and metabolic fitness of armored CAR-NK cells with IL-15 secretion could be enhanced by targeting a cytokine checkpoint, which represented an important milestone in the exploration of the next-generation cancer immunotherapy [41, 117, 164, 165]. Another study conducted combined CAR-T infusion after NKG2D.ζ-NK cell administration and found improved anti-cancer activity and tumor infiltration [55]. Collectively, the pioneering preclinical and clinical studies have suggested the multifaceted opportunities and challenges of allogeneic CAR-NK cells, which are recognized as pivotal and promising “off-the-shelf” product in the next-generation cellular immunotherapies targeting recurrent and refractory malignancies.

## Data Availability

All data are included in this published article. Meanwhile, the datasets involved in the current study are available from the corresponding author on reasonable request.

## Abbreviations

CAR-NK: Chimeric antigen receptor-transduced NK
CRS: Cytokine release syndrome
GVHD: Graft-versus-host disease
irAEs: Immune-related adverse events
CAR-T: Chimeric antigen receptor-modified T
TCR-T: T cell receptor-engineered T
TILs: Tumor-infiltrating lymphocytes
APCs: Antigen-presenting cells
ADCC: Antibody-dependent cell-mediated cytotoxicity
AML: Acute myeloid leukemia
LSCs: Leukemia stem cells
PB-NK: Peripheral blood-derived NK
Bcp-ALL: B-cell precursor acute lymphocytic leukemia
UC-NK: Umbilical cord blood-derived NK
P-NK: Placental blood-derived NK
hPSCs: Human pluripotent stem cells
hiPSCs: Human induced pluripotent stem cells
hESCs: Human embryonic stem cells
MSCs: Mesenchymal stem/stromal cells
EpCAM: Epithelial cell adhesion molecule
ICANS: Immune cell- associated neurotoxicity syndrome
GMP: Good manufacturing practice
NCRs: Natural cytotoxicity receptors
